# 4-Nitro­benzyl 2-bromo­acetate

**DOI:** 10.1107/S1600536809021187

**Published:** 2009-06-06

**Authors:** Kai Zhu, Hui Liu, Yan-Hua Wang, Ping-Fang Han, Ping Wei

**Affiliations:** a College of Biotechnology and Pharmaceutical Engineering, Nanjing University of Technolgy, Xinmofan Road No. 5 Nanjing, Nanjing 210009, People’s Republic of China

## Abstract

In the mol­ecule of the title compound, C_9_H_8_BrNO_4_, the acetate group is close to planar [maximum deviation = 0.042 (3) Å] and is oriented at a dihedral angle of 73.24 (3)° with respect to the aromatic ring. In the crystal structure, inter­molecular C—H⋯O inter­actions link the mol­ecules into a three-dimensional network, forming *R*_2_^2^(10) ring motifs.

## Related literature

For a related structure, see: Pyun *et al.* (2001 ▶). For bond-length data, see: Allen *et al.* (1987 ▶). For ring motifs, see: Bernstein *et al.* (1995 ▶).
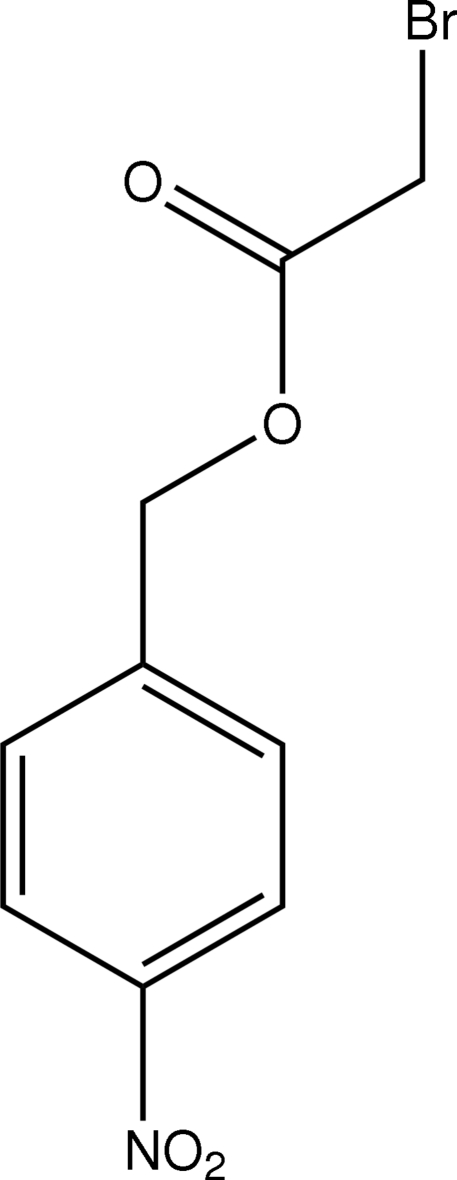


## Experimental

### 

#### Crystal data

C_9_H_8_BrNO_4_
*M* *_r_* = 274.07
Monoclinic, 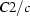

*a* = 13.851 (3) Å
*b* = 8.1590 (16) Å
*c* = 19.201 (4) Å
β = 109.08 (3)°
*V* = 2050.7 (8) Å^3^
*Z* = 8
Mo *K*α radiation
μ = 4.00 mm^−1^
*T* = 294 K
0.20 × 0.10 × 0.10 mm

#### Data collection

Enraf–Nonius CAD-4 diffractometer
Absorption correction: ψ scan (North *et al.*, 1968 ▶) *T*_min_ = 0.502, *T*_max_ = 0.690
3739 measured reflections
1873 independent reflections
1055 reflections with *I* > 2σ(*I*)
*R*_int_ = 0.043
3 standard reflections frequency: 120 min intensity decay: 1%

#### Refinement

*R*[*F* ^2^ > 2σ(*F* ^2^)] = 0.054
*wR*(*F* ^2^) = 0.131
*S* = 1.00
1873 reflections
136 parameters
H-atom parameters constrained
Δρ_max_ = 0.49 e Å^−3^
Δρ_min_ = −0.46 e Å^−3^

### 

Data collection: *CAD-4 Software* (Enraf–Nonius, 1989 ▶); cell refinement: *CAD-4 Software*; data reduction: *XCAD4* (Harms & Wocadlo, 1995 ▶); program(s) used to solve structure: *SHELXS97* (Sheldrick, 2008 ▶); program(s) used to refine structure: *SHELXL97* (Sheldrick, 2008 ▶); molecular graphics: *ORTEP-3 for Windows* (Farrugia, 1997 ▶) and *PLATON* (Spek, 2009 ▶); software used to prepare material for publication: *SHELXL97* and *PLATON*.

## Supplementary Material

Crystal structure: contains datablocks global, I. DOI: 10.1107/S1600536809021187/hk2706sup1.cif

Structure factors: contains datablocks I. DOI: 10.1107/S1600536809021187/hk2706Isup2.hkl

Additional supplementary materials:  crystallographic information; 3D view; checkCIF report

## Figures and Tables

**Table 1:** Hydrogen-bond geometry (Å, °)

*D*—H⋯*A*	*D*—H	H⋯*A*	*D*⋯*A*	*D*—H⋯*A*
C7—H7*A*⋯O4^i^	0.97	2.59	3.486 (7)	153
C9—H9*B*⋯O4^ii^	0.97	2.47	3.376 (8)	155

Symmetry codes: (i) 
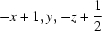
; (ii) 
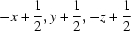
.

**Fig. 1.**
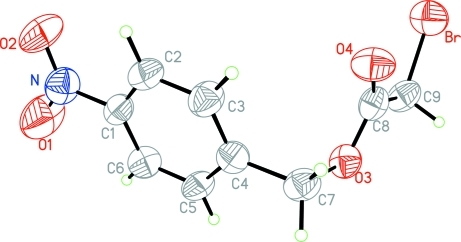
The molecular structure of the title molecule, with the atom-numbering scheme. Displacement ellipsoids are drawn at the 30% probability level.

**Fig. 2.**
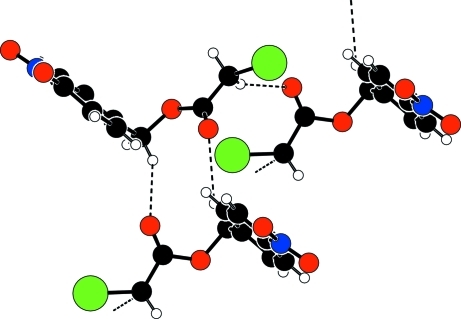
A partial packing diagram of the title compound. Hydrogen bonds are shown as dashed lines.

## supplementary crystallographic information

### Comment

Some derivatives of p-nitrobenzyl alcohol are important chemical materials. We report herein the crystal structure of the title compound.

In the molecule of the title compound (Fig 1), the bond lengths (Allen *et al.*,  1987) and angles are within normal ranges. Ring A (C1-C6) is, of course, planar. Atoms O1, O2, N, and C7 are 0.119 (3), -0.161 (3), -0.015 (3) and -0.042 (3) Å away from the plane of ring A, respectively. The moiety (O3/O4/C7-C9) is planar with a maximum deviation of -0.042 (3) Å for atom C7, and it is oriented with respect to ring A at a dihedral angle of 73.24 (3)°.

In the crystal structure, intermolecular C-H···O interactions (Table 1) link the molecules into a three-dimensional network forming *R*_2_^2^(10) ring motifs (Bernstein *et al.*, 1995) (Fig. 2), in which they may be effective in the stabilization of the structure.

### Experimental

For the preparation of the title compound, bromoacetyl bromide (2.01 g) and p-nitrobenzyl alcohol (1.53 g) were added into dichloromethane (30 ml) in pyridine (15 ml) at 273-278 K. The gross products were extracted with n-hexane, washed with water, dried under vaccum, and then recrystallized from dichloromethane (yield; 0.503 g) (Pyun *et al.*, 2001). Crystals suitable for X-ray analysis were obtained by slow evaporation of a methanol solution.

### Refinement

H atoms were positioned geometrically, with C-H = 0.93 and 0.97 Å for aromatic and methylene H, respectively, and constrained to ride on their parent atoms, with U_iso_(H) = 1.2U_eq_(C).

### Crystal data

**Table:**

C_9_H_8_BrNO_4_	*F*(000) = 1088
*M**_r_* = 274.07	*D*_x_ = 1.775 Mg m^−^^3^
Monoclinic, *C*2/*c*	Mo *K*α radiation, λ = 0.71073 Å
Hall symbol: -C 2yc	Cell parameters from 25 reflections
*a* = 13.851 (3) Å	θ = 10–14°
*b* = 8.1590 (16) Å	µ = 4.00 mm^−^^1^
*c* = 19.201 (4) Å	*T* = 294 K
β = 109.08 (3)°	Block, colorless
*V* = 2050.7 (8) Å^3^	0.20 × 0.10 × 0.10 mm
*Z* = 8	

### Data collection

**Table:**

Enraf–Nonius CAD-4 diffractometer	1055 reflections with *I* > 2σ(*I*)
Radiation source: fine-focus sealed tube	*R*_int_ = 0.043
graphite	θ_max_ = 25.3°, θ_min_ = 2.2°
ω/2θ scans	*h* = 0→16
Absorption correction: ψ scan (North *et al.*, 1968)	*k* = −9→9
*T*_min_ = 0.502, *T*_max_ = 0.690	*l* = −23→21
3739 measured reflections	3 standard reflections every 120 min
1873 independent reflections	intensity decay: 1%

### Refinement

**Table:**

Refinement on *F*^2^	Primary atom site location: structure-invariant direct methods
Least-squares matrix: full	Secondary atom site location: difference Fourier map
*R*[*F*^2^ > 2σ(*F*^2^)] = 0.054	Hydrogen site location: inferred from neighbouring sites
*wR*(*F*^2^) = 0.131	H-atom parameters constrained
*S* = 1.00	*w* = 1/[σ^2^(*F*_o_^2^) + (0.044*P*)^2^] where *P* = (*F*_o_^2^ + 2*F*_c_^2^)/3
1873 reflections	(Δ/σ)_max_ < 0.001
136 parameters	Δρ_max_ = 0.49 e Å^−^^3^
0 restraints	Δρ_min_ = −0.46 e Å^−^^3^

### Special details

**Table:**

Geometry. All e.s.d.'s (except the e.s.d. in the dihedral angle between two l.s. planes) are estimated using the full covariance matrix. The cell e.s.d.'s are taken into account individually in the estimation of e.s.d.'s in distances, angles and torsion angles; correlations between e.s.d.'s in cell parameters are only used when they are defined by crystal symmetry. An approximate (isotropic) treatment of cell e.s.d.'s is used for estimating e.s.d.'s involving l.s. planes.
Refinement. Refinement of *F*^2^ against ALL reflections. The weighted *R*-factor *wR* and goodness of fit *S* are based on *F*^2^, conventional *R*-factors *R* are based on *F*, with *F* set to zero for negative *F*^2^. The threshold expression of *F*^2^ > σ(*F*^2^) is used only for calculating *R*-factors(gt) *etc*. and is not relevant to the choice of reflections for refinement. *R*-factors based on *F*^2^ are statistically about twice as large as those based on *F*, and *R*- factors based on ALL data will be even larger.

### Fractional atomic coordinates and isotropic or equivalent isotropic displacement parameters (Å^2^)

**Table:**

	*x*	*y*	*z*	*U*_iso_*/*U*_eq_	
Br	0.14001 (5)	0.10006 (10)	0.25541 (4)	0.0690 (3)	
O1	0.3414 (6)	−0.3704 (8)	−0.1100 (3)	0.114 (2)	
O2	0.3855 (4)	−0.5164 (7)	−0.0136 (3)	0.0823 (16)	
O3	0.3187 (3)	0.2414 (5)	0.1386 (2)	0.0474 (10)	
O4	0.3549 (3)	0.0826 (5)	0.2389 (2)	0.0550 (11)	
N	0.3685 (4)	−0.3840 (8)	−0.0450 (4)	0.0628 (16)	
C1	0.3826 (4)	−0.2328 (8)	0.0002 (3)	0.0436 (15)	
C2	0.4247 (4)	−0.2437 (8)	0.0752 (3)	0.0485 (16)	
H2A	0.4448	−0.3443	0.0980	0.058*	
C3	0.4364 (5)	−0.1024 (8)	0.1156 (3)	0.0508 (15)	
H3A	0.4656	−0.1081	0.1666	0.061*	
C4	0.4064 (4)	0.0471 (8)	0.0832 (3)	0.0428 (15)	
C5	0.3638 (4)	0.0536 (8)	0.0067 (3)	0.0482 (16)	
H5A	0.3430	0.1538	−0.0164	0.058*	
C6	0.3524 (4)	−0.0851 (8)	−0.0343 (3)	0.0515 (17)	
H6A	0.3243	−0.0801	−0.0854	0.062*	
C7	0.4165 (4)	0.1993 (8)	0.1284 (3)	0.0483 (15)	
H7A	0.4681	0.1828	0.1762	0.058*	
H7B	0.4384	0.2893	0.1041	0.058*	
C8	0.2977 (4)	0.1691 (8)	0.1950 (3)	0.0429 (14)	
C9	0.1933 (4)	0.2229 (8)	0.1920 (3)	0.0540 (16)	
H9A	0.1474	0.2124	0.1417	0.065*	
H9B	0.1957	0.3377	0.2055	0.065*	

### Atomic displacement parameters (Å^2^)

**Table:**

	*U*^11^	*U*^22^	*U*^33^	*U*^12^	*U*^13^	*U*^23^
Br	0.0593 (4)	0.0744 (5)	0.0828 (5)	0.0128 (4)	0.0362 (4)	0.0107 (5)
O1	0.189 (6)	0.076 (5)	0.065 (3)	0.000 (5)	0.028 (4)	−0.022 (3)
O2	0.102 (4)	0.036 (3)	0.100 (4)	−0.008 (3)	0.021 (3)	−0.004 (3)
O3	0.044 (2)	0.045 (3)	0.051 (2)	0.010 (2)	0.0132 (18)	0.003 (2)
O4	0.044 (2)	0.056 (3)	0.061 (2)	0.010 (2)	0.012 (2)	0.020 (3)
N	0.059 (3)	0.056 (5)	0.070 (4)	−0.008 (3)	0.018 (3)	−0.011 (4)
C1	0.033 (3)	0.037 (4)	0.060 (4)	0.000 (3)	0.015 (3)	−0.002 (3)
C2	0.049 (3)	0.039 (4)	0.055 (4)	0.004 (3)	0.014 (3)	0.007 (3)
C3	0.057 (3)	0.044 (4)	0.047 (3)	0.005 (4)	0.011 (3)	0.002 (4)
C4	0.029 (3)	0.046 (4)	0.054 (4)	−0.003 (3)	0.015 (3)	−0.002 (3)
C5	0.054 (4)	0.035 (4)	0.052 (4)	0.005 (3)	0.012 (3)	0.008 (3)
C6	0.049 (4)	0.053 (4)	0.047 (3)	0.001 (3)	0.008 (3)	0.003 (4)
C7	0.040 (3)	0.044 (4)	0.060 (4)	−0.004 (3)	0.017 (3)	0.001 (4)
C8	0.032 (3)	0.042 (4)	0.050 (3)	−0.006 (3)	0.008 (3)	−0.010 (3)
C9	0.046 (3)	0.041 (4)	0.075 (4)	0.005 (3)	0.019 (3)	0.001 (4)

### Geometric parameters (Å, °)

**Table:**

Br—C9	1.903 (6)	C3—H3A	0.9300
O3—C7	1.470 (6)	C4—C5	1.393 (8)
O3—C8	1.346 (7)	C4—C7	1.496 (8)
O4—C8	1.183 (7)	C5—C6	1.359 (8)
N—O1	1.185 (7)	C5—H5A	0.9300
N—O2	1.222 (7)	C6—H6A	0.9300
N—C1	1.485 (8)	C7—H7A	0.9700
C1—C2	1.367 (8)	C7—H7B	0.9700
C1—C6	1.373 (8)	C8—C9	1.494 (8)
C2—C3	1.370 (8)	C9—H9A	0.9700
C2—H2A	0.9300	C9—H9B	0.9700
C3—C4	1.371 (8)		
			
C8—O3—C7	117.1 (5)	C5—C6—C1	119.4 (5)
O1—N—O2	123.1 (7)	C5—C6—H6A	120.3
O1—N—C1	118.3 (7)	C1—C6—H6A	120.3
O2—N—C1	118.6 (6)	O3—C7—C4	110.8 (4)
C2—C1—C6	121.6 (6)	O3—C7—H7A	109.5
C2—C1—N	119.4 (6)	C4—C7—H7A	109.5
C6—C1—N	119.0 (6)	O3—C7—H7B	109.5
C1—C2—C3	118.2 (6)	C4—C7—H7B	109.5
C1—C2—H2A	120.9	H7A—C7—H7B	108.1
C3—C2—H2A	120.9	O4—C8—O3	124.3 (5)
C2—C3—C4	121.9 (5)	O4—C8—C9	128.1 (6)
C2—C3—H3A	119.0	O3—C8—C9	107.5 (6)
C4—C3—H3A	119.0	C8—C9—Br	113.2 (5)
C3—C4—C5	118.3 (6)	C8—C9—H9A	108.9
C3—C4—C7	121.2 (5)	Br—C9—H9A	108.9
C5—C4—C7	120.5 (6)	C8—C9—H9B	108.9
C6—C5—C4	120.6 (6)	Br—C9—H9B	108.9
C6—C5—H5A	119.7	H9A—C9—H9B	107.8
C4—C5—H5A	119.7		
			
O1—N—C1—C2	−172.7 (6)	C4—C5—C6—C1	0.5 (9)
O2—N—C1—C2	7.3 (8)	C2—C1—C6—C5	−0.5 (9)
O1—N—C1—C6	7.6 (9)	N—C1—C6—C5	179.2 (5)
O2—N—C1—C6	−172.4 (6)	C8—O3—C7—C4	85.8 (6)
C6—C1—C2—C3	−0.1 (8)	C3—C4—C7—O3	−98.5 (6)
N—C1—C2—C3	−179.8 (5)	C5—C4—C7—O3	79.9 (6)
C1—C2—C3—C4	0.6 (9)	C7—O3—C8—O4	4.6 (8)
C2—C3—C4—C5	−0.6 (9)	C7—O3—C8—C9	−177.1 (5)
C2—C3—C4—C7	177.8 (5)	O4—C8—C9—Br	−13.4 (8)
C3—C4—C5—C6	0.1 (8)	O3—C8—C9—Br	168.3 (4)
C7—C4—C5—C6	−178.4 (5)		

### Hydrogen-bond geometry (Å, °)

**Table:**

*D*—H···*A*	*D*—H	H···*A*	*D*···*A*	*D*—H···*A*
C7—H7A···O4^i^	0.97	2.59	3.486 (7)	153
C9—H9B···O4^ii^	0.97	2.47	3.376 (8)	155

Symmetry codes: (i) −*x*+1, *y*, −*z*+1/2; (ii) −*x*+1/2, *y*+1/2, −*z*+1/2.
